# Reconceptualizing synergism and antagonism among multiple stressors

**DOI:** 10.1002/ece3.1465

**Published:** 2015-03-11

**Authors:** Jeremy J Piggott, Colin R Townsend, Christoph D Matthaei

**Affiliations:** Department of Zoology, University of OtagoP.O. Box 56, Dunedin, 9054, New Zealand

**Keywords:** Antagonism, ecological surprise, interaction, stressor, synergism

## Abstract

The potential for complex synergistic or antagonistic interactions between multiple stressors presents one of the largest uncertainties when predicting ecological change but, despite common use of the terms in the scientific literature, a consensus on their operational definition is still lacking. The identification of synergism or antagonism is generally straightforward when stressors operate in the same direction, but if individual stressor effects oppose each other, the definition of synergism is paradoxical because what is synergistic to one stressor's effect direction is antagonistic to the others. In their highly cited meta-analysis, Crain et al. (*Ecology Letters*, 11, 2008: 1304) assumed in situations with opposing individual effects that synergy only occurs when the cumulative effect is more negative than the additive sum of the opposing individual effects. We argue against this and propose a new systematic classification based on an additive effects model that combines the magnitude and response direction of the cumulative effect and the interaction effect. A new class of “mitigating synergism” is identified, where cumulative effects are reversed and enhanced. We applied our directional classification to the dataset compiled by Crain et al. (*Ecology Letters,* 11, 2008: 1304) to determine the prevalence of synergistic, antagonistic, and additive interactions. Compared to their original analysis, we report differences in the representation of interaction classes by interaction type and we document examples of mitigating synergism, highlighting the importance of incorporating individual stressor effect directions in the determination of synergisms and antagonisms. This is particularly pertinent given a general bias in ecology toward investigating and reporting adverse multiple stressor effects (double negative). We emphasize the need for reconsideration by the ecological community of the interpretation of synergism and antagonism in situations where individual stressor effects oppose each other or where cumulative effects are reversed and enhanced.

## Introduction

The potential for complex synergistic or antagonistic interactions between multiple stressors presents one of the largest uncertainties when predicting ecological change (Sala et al. 2000; MEA 2005; Mothersill et al. 2007; Darling and Cote 2008). Despite common use of “synergism” in the scientific literature, a consensus on its operational definition when classifying interactive effects is still lacking (Berenbaum 1989; Folt et al. 1999; Chou 2010; Dunne 2010; Vanhoudt et al. 2012). In the ecological multiple stressor context, the term most commonly relates to an additive effects model. Thus, synergism is used to define a cumulative effect of multiple stressors that are greater than the additive sum of effects produced by the stressors acting in isolation; this contrasts with the term “antagonism,” used to define a cumulative effect that is less than additive (Hay et al. 1994; Hay 1996; Folt et al. 1999).

In ecological research, the term “stressor” has frequently been used synonymously with “pollution,” “pollutants,” or “pressures” on the assumption that the effects of a stressor imply “stress” and must therefore be exclusively detrimental (Folt et al. 1999). However, what is stressful or detrimental to one species in an ecosystem is likely to be beneficial to another, either directly or via species interactions. Moreover, stressor responses may follow a subsidy-stress gradient, as observed, for example, for stream taxa in relation to dissolved nutrient concentration (Niyogi et al. 2007). Therefore, we define a stressor as a variable that, as a result of human activity, exceeds its range of normal variation and affects (whether negatively or positively) individual taxa, community composition, or ecosystem functioning relative to a reference condition (e.g., modified after Breitburg et al. 1999a; Crain et al. 2008; Townsend et al. 2008).

**Figure 1:**
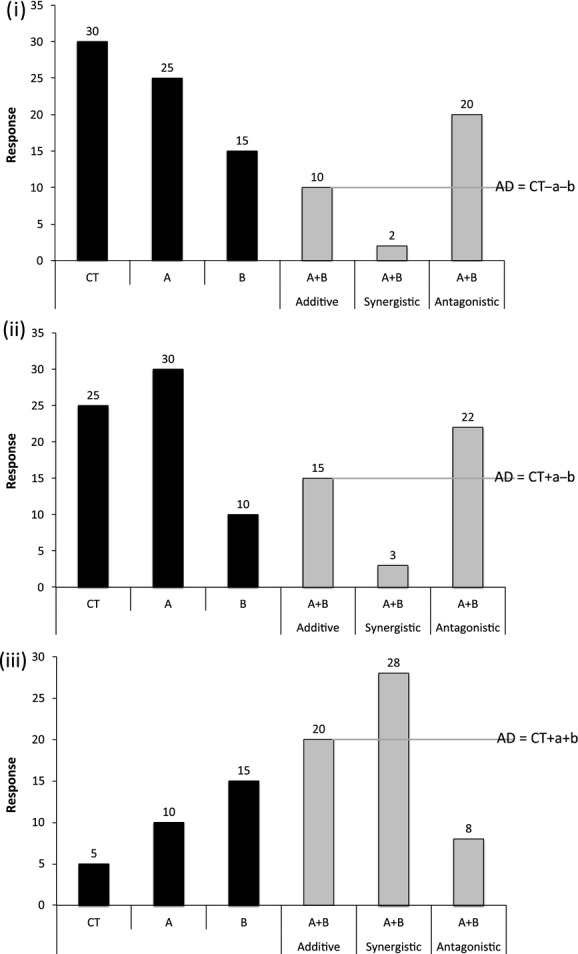
Redrawn from Crain et al. (2008). Conceptual approach to interpreting interaction types from response data presented in factorial studies. Treatments in factorial studies include control (CT), with stressor A (A), with stressor B (B), and with both stressors (A + B). Interaction types are classified as additive, synergistic, and antagonistic, depending on the A + B response compared to the additive sum (AD) of individual effects for stressor A (a), B (b) relative to the control (CT). The three plots show interaction types that have double-negative (i), opposing (ii), and double-positive (iii) individual stressor effects on the response variable of interest.

In their highly cited synthesis of 170 studies manipulating pairs of stressors in marine and coastal ecosystems, Crain et al. (2008) conceptualized three broad categories of interaction type based on the directions of individual stressor effects: The two individual stressors operate negatively (double negative), positively (double positive), or with opposing (one positive and one negative) individual effects relative to control conditions (Fig.1). While the identification of a synergism or antagonism is generally straightforward when both stressors operate in the same direction (i.e., double positive or double negative; Folt et al. 1999; Dunne 2010), for opposing individual effects, the definition of synergism seems paradoxical because what is synergistic to one stressor's effect direction is antagonistic to the other stressor's effect direction and vice versa. Note that from a purely mathematical perspective, this is not a paradox because synergy can be defined in either direction determined by a larger positive or negative cumulative effect relative to the individual stressor effects in absolute terms. Given the lack of consensus regarding these terms, Crain et al. (2008) assumed that in situations where two individual stressors oppose each other, synergy only occurs when the cumulative effect is more negative than the additive sum of the opposing individual effects (see Fig. 1ii). While this may be appropriate in situations where the effect direction is implicitly negative (e.g., decreased survival rate), such a definition is problematic from an ecological perspective because effect direction is entirely context dependent. Take the example of a data set for decomposition of leaf matter where nutrient enrichment alone accelerates decay while sediment addition alone slows decay, but both stressors in combination cause a decay rate even greater than with nutrient enrichment alone. This interactive pattern of leaf decay could be presented either positively (as rate of leaf mass loss) or negatively (as leaf mass remaining). Depending on which perspective was assumed in the analysis, one could conclude synergism for leaf mass remaining or antagonism for rate of loss, yet the interaction is clearly synergistic (as measured by the magnitude of the cumulative effect).

The assumption of synergy when the cumulative effect is more negative than the additive sum of the opposing individual effects raises a further conceptual issue, because this pattern does not necessarily mean the cumulative effect of the opposing stressors is more negative than the single negative stressor acting alone (equivalent to the “comparative effects” model of Folt et al. 1999). This can be illustrated by the following theoretical example: a positive stressor individually has an effect of +1, a negative stressor individually has an effect of −1, and the additive cumulative effect of both stressors combined is 0 (i.e., they counteract each other). In this situation, Crain et al. (2008) would invoke synergy for any cumulative effect more negative than 0. But if the cumulative effect is between −1 and 0, this outcome is intuitively antagonistic from the perspective of the negative stressor's individual effect (i.e., the cumulative effect of both stressors is less negative than the single negative stressor acting alone).

In toxicology, in contrast to ecological usage, if one chemical is rendered more effective by the presence of another that has no effect or a different effect on its own, the interaction is called potentiation or sensitization (Odum and Barrett 2005). Chou (2010) argues that this interaction type is not a true synergism because it is “one-sided” and the underlying modes of action are different. If this perspective was to be generally accepted, the term synergy would only apply when both stressors operate in the same direction.

To resolve the issues raised above (stressor effects may be detrimental or beneficial, difficulty in defining synergy when individual effects are opposing), we believe an alternative system is needed that systematically classifies synergisms in any directional context as measured by the magnitude of the cumulative effect compared to the individual stressor effects.

### A new directional interaction classification for ecological data

**Figure 2:**
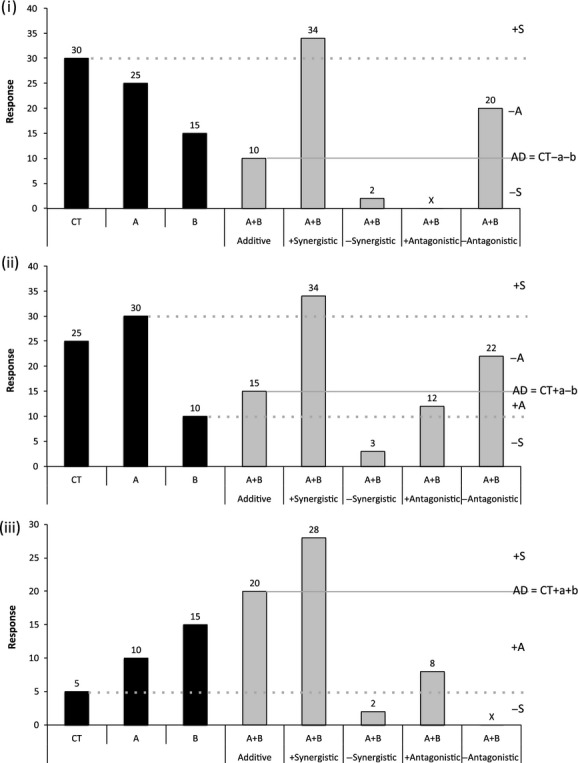
Our conceptual approach to interpreting interaction types from data presented in factorial studies determined from the magnitude and direction of the cumulative effect and interaction effect in absolute terms. Treatments in factorial studies include control (CT), with stressor A (A), with stressor B (B), and with both stressors (A + B). Directional interaction classes are additive (AD), +synergistic (+S), −synergistic (−S), +antagonistic (+A) and -antagonistic (−A) that vary depending on A + B compared to the additive sum (AD) of individual effects for stressor A (a), B (b) relative to the control (CT). The three plots show interaction types that have double-negative (i), opposing (ii), and double-positive (iii) individual stressors effects on the response variable of interest. (X) indicates that an interaction class is not applicable for the interaction type in question. Figure based on a reanalysis of the database of Crain et al. (2008).

We propose a classification system based on an additive effects model that combines the magnitude and response direction (+ or −) of the cumulative effect (effect of combined stressors relative to control) and interaction effect (effect deviation from the additive model prediction) to determine synergism and antagonism relative to individual stressor effects in absolute terms (Fig.2). Selection of an additive effects model is consistent with the prevailing definition of synergy and the use of ANOVA as the statistical analysis of choice for response data presented in factorial studies (Quinn and Keough 2002; Dunne 2010). In this directional classification system, the meanings of the words synergism and antagonism respectively translate to “more-than” or “less-than” predicted additively in absolute terms (i.e., with the stated direction; Piggott et al. 2015a,b).

1. An *additive effect* (i.e., no significant interaction in ANOVA) represents the sum of the individual effects that may arise from double-positive effects (e.g., +1 + 1 = 2 for an effect of one for each stressor), double-negative effects (−1 + −1 = −2), or opposing effects (−1 + 1 = 0).
2. An interaction that deviates from additive (i.e., a significant two-factor interaction term in ANOVA) and is less than the sum of the individual effects or less-than-or-equal-to any individual effect in the same direction is *positive antagonistic* (+A; less positive than predicted additively) when +1 + 1 = 0 < (+A) < 2 or −1 + 1 = −1 ≤ (+A) < 0, or *negative antagonistic* (−A; less negative than predicted additively) when −1 + −1 = −2 < (−A)<0 or −1 + 1 = 0 < (−A) ≤ 1.
3. A deviation from additive that is greater than the sum of individual effects and greater than any individual effect in the same direction or has an interaction effect that is greater than both in absolute terms is *positive synergistic* (+S; more positive than predicted additively) when +1 + 1 = (+S) > 2 or −1 + −1 = (+S) > 0 or −1 + 1 = (+S) > 1, or *negative synergistic* (−S; more negative than predicted additively) when +1 + 1 = (−S) < 0 or −1 + −1 = (−S) < −2 or −1 + 1 = (−S) < −1.

Table1 presents directional classifications for all potential two-factor interaction types that may theoretically occur in addition to those stated above.

**Table 1:** Potential interaction types and directional classifications for two-variable response data in factorial studies. The direction of individual stressor effects (a) or (b) and interaction effect (a + b) are coded as positive (+), negative (−), or neutral (0). Double-sign symbols (++) or (––) indicate the direction of a cumulative effect (a + b) that is greater than the sum of individual effects and greater than any individual effect in the same direction or has an interaction effect that is greater than both in absolute terms. Directional interaction classes are additive (AD), +synergistic (+S), −synergistic (−S), +antagonistic (+A), and –antagonistic (−A)

Interaction Type	a	b	a + b	Classification
Double positive	+	+	++	+S
	+	+	+	+S
	+	+	0	AD
	+	+	−	+A
	+	+	––	−S
Double negative	−	−	++	+S
	−	−	+	−A
	−	−	0	AD
	−	−	−	−S
	−	−	––	−S
Opposing	+	−	++	+S
	+	−	+	−A
	+	−	0	AD
	+	−	−	+A
	+	−	–	−S
Opposing	−	+	++	+S
	−	+	+	−A
	−	+	0	AD
	−	+	−	+A
	−	+	––	−S
Negative Neutral	−	0	++	+S
	−	0	+	−A
	−	0	0	AD
	−	0	−	−S
	−	0	––	−S
Negative Neutral	0	−	++	+S
	0	−	+	−A
	0	−	0	AD
	0	−	−	−S
	0	−	––	−S
Positive Neutral	+	0	++	+S
	+	0	+	+S
	+	0	0	AD
	+	0	−	+A
	+	0	––	−S
Positive Neutral	0	+	++	+S
	0	+	+	+S
	0	+	0	AD
	0	+	−	+A
	0	+	––	−S
Double Neutral	0	0	++	+S
	0	0	+	+S
	0	0	0	AD
	0	0	−	−S
	0	0	––	−S

### Assessing the directional classification using Crain et al.'s dataset

To assess the applicability of our directional classification approach to ecological inquiry, we applied it to the dataset compiled by Crain et al. (2008). Following their methodology, we inferred additive cumulative effects where the 95% confidence intervals of the Hedge's *d* interaction effect overlapped zero (i.e., effect summation; Gurevitch et al. 2000). We then applied our directional classification to nonadditive interactions based on interaction type (Table1). In situations where individual stressor effects opposed each other or where large interaction effects were present, we inferred synergism only in situations where the 95% confidence intervals of the cumulative effect Hedge's *d* did not overlap the Hedge's *d* effect values of the individual stressors or the control (refer to Crain et al. 2008 for details).

The 170 multiple stressor studies yielded examples of all six potential interaction types set out in Table1. Double-negative (41%) interactions were most frequent, while opposing (29%) and double-positive interactions (22%) were also common. Negative neutral (5%) interactions were less frequent, and positive neutral (2%) and double-neutral (1%) interactions were rare (Table2; Fig. S1). Overall, interactions (regardless of direction) were most often antagonistic (43%), but also frequently synergistic (31%) or additive (26%) (Table2). Applying a directional orientation to all nonadditive effects revealed that negative antagonisms (28%) and negative synergisms (24%) were the most common nonadditive interaction classes, whereas positive antagonisms (15%) were less frequent and positive synergisms (7%) least common. Frequencies of interaction classes varied markedly by interaction type. Double-negative interactions (70 instances) were less negative than predicted additively in more than half of all instances (negative antagonistic; 51%), more negative than predicted in about a quarter (negative synergistic; 26%), and as predicted in roughly a fifth of all instances (additive; 21%). In contrast, double-positive interactions (38 instances) were as predicted in over a third of all instances (additive; 37%) or less positive than predicted in just under a third (positive antagonistic; 29%), with roughly a quarter yielding positive synergistic cumulative effects (24%) and in four instances becoming negative (negative synergistic; 11%). Opposing interactions (49 instances) were fairly evenly distributed between positive antagonistic (27%), additive (24%), negative antagonistic (22%), and negative synergistic (20%) cumulative effects, with positive synergistic (6%) outcomes least frequent. Negative neutral interactions (nine instances) resulted in enhanced negative effects (negative synergistic; 67%) in two-thirds of instances but were unchanged in a third (additive; 33%). Positive neutral interactions (three instances) tended to be less positive with the addition of a neutral stressor (positive antagonistic; 67%) or became negative (negative synergistic; 33%). Lastly, the only double-neutral interaction in the dataset yielded a negative cumulative effect (negative synergistic; 100%).

**Table 2:** Frequencies and percentages of interaction classes by interaction type summarizing 170 studies manipulating two or more stressors in marine and coastal systems reclassified from Crain et al. (2008). Directional interaction classes are additive (AD), +synergistic (+S), −synergistic (−S), +antagonistic (+A), and –antagonistic (−A). (X) indicates an interaction class is not applicable for the interaction type. Full details of each study/interaction are given in the Table S1

Interaction Type	Classification	Frequency	%
Double negative	AD	15	21
(70 instances)	−S	19	27
(41% of Total)	+S	0	0
	−A	36	51
	+A	X	X
Double positive	AD	14	37
(38 instances)	−S	4	11
(22% of Total)	+S	9	24
	−A	X	X
	+A	11	29
Opposing	AD	12	24
(49 instances)	−S	10	20
(29% of Total)	+S	3	6
	−A	11	22
	+A	13	27
Negative neutral	AD	3	33
(nine instances)	−S	6	67
(5% of Total)	+S	0	0
	−A	0	0
	+A	X	X
Positive neutral	AD	0	0
(three instances)	−S	1	33
(2% of Total)	+S	0	0
	−A	X	X
	+A	2	67
Double neutral	AD	0	0
(one instance)	−S	1	100
(1% of Total)	+S	0	0
	−A	X	X
	+A	X	X
Total	AD	44	26
(170 instances)	−S	41	24
(100%)	+S	12	7
	−A	47	28
	+A	26	15
Total (w/o direction)	Synergism	53	31
(170 instances)	Antagonism	73	43
(100%)	Additive	44	26

### New classes of interaction – “positive synergisms” and “mitigating synergisms”

In contrast to Crain et al. (2008), our classification approach includes a new class of positive synergism in situations where effects are opposing (+S in “Opposing” rows of Table1). Moreover, our system has the merit of providing additional information about the directional nature of each interaction. A potentially controversial aspect of the system is the inclusion of a further new class, which we call “mitigating synergism.” In this class, two stressors operating in the same direction create a cumulative effect completely opposite to what would have been predicted; that is, two positives make a negative (−S in “Double positive” row of Table1) or two negatives make a positive (+S in “Double negative” row). Some might argue that such a pattern is the clearest example of antagonism because individual effects are reversed. However, classifying this interaction as antagonistic (according to the currently prevalent definition of this term in the ecological literature) would be misleading in our view because the magnitude of the interaction effect is greater than one would predict based on the two single stressor effects in absolute terms. Moreover, such strong interactions may have the most interesting ecological consequences because they suggest that when both stressors act together, they synergistically mitigate or inhibit their individual effects even more than under control conditions (i.e., effect reversal with enhancement). In epidemiological research, such so-called crossover interactions are considered the most statistically robust class of interaction because they indicate that risk factors flip from being disease predisposing in one background to protective in another (Kendler and Gardner 2010). There is, however, deep controversy surrounding the expected prevalence of such interactions between epidemiologists (Kendler and Gardner 2010).

A recent review of the interacting roles of stressors driving the global loss of canopy-forming to mat-forming algae in marine ecosystems by Strain et al. (2014) utilized a directional interaction classification that documented examples of mitigating synergisms, although they did not use the term. However, their approach neglected to distinguish antagonism in situations where stressor effects were opposing.

### Comparing the results of our classification with that of Crain et al

When applying our directional classifications to the dataset compiled by Crain et al. (2008), we found examples of each potential interaction type and class, although class representation varied by interaction type. Of particular note were four instances of mitigating synergism that occurred in double-positive interactions (Breitburg et al. 1999b; Przeslawski et al. 2005; Sargian et al. 2007; Swanson and Fox 2007), indicating that two positive stressors can indeed produce a negative cumulative effect. Similarly, effect reversal and enhancement was observed in a positive neutral interaction (Pelletier et al. 2006), suggesting that agents that have no discernible effect individually may catalyze inhibitory or mitigating responses to other stressor effects. These examples involved the following stressor pairs: nutrient and toxin (Breitburg et al. 1999b), CO_2_ and UV (Swanson and Fox 2007), salinity and temperature (Przeslawski et al. 2005), and toxin and UV (Pelletier et al. 2006; Sargian et al. 2007). Toxin and UV also had no discernible individual effects in the only double-neutral interaction that resulted in a negative cumulative effect (i.e., negative synergism; Pelletier et al. 2006). Considering the numerous direct and indirect pathways along which these physicochemical stressors may propagate through ecosystems, complex outcomes are perhaps not surprising. While we found no evidence in the dataset compiled by Crain et al. (2008) of two negative stressors producing a positive cumulative effect (the second type of mitigating synergism defined above), such outcomes are not unheard of. For example, Christensen et al. (2006) observed a positive synergistic interaction for consumer biomass between raised temperature, drought-induced UVB exposure and acidity in temperate lakes, despite each stressor individually exerting negative effects. Our classification approach, with its identification of such instances, is a first step toward elucidating the mechanisms behind such complex patterns and inferring generality of effects.

Of the 49 opposing interactions classified by Crain et al. (2008), we reclassified three antagonisms as positive synergisms (enhanced positive effect when negative stressor present) and eleven synergisms as positive antagonisms (less positive than predicted additively, but less negative than the negative stressor alone). Thus, Crain et al.'s (2008) conceptualization of synergism and antagonism for interactions involving stressors with opposing individual effects underrepresented positive synergisms (because they were not possible) but overrepresented negative synergisms. Together with our reclassification of four double-positive antagonisms as negative synergisms, this accounts for the discrepancy in the (direction-independent) prevalence of interaction classes between our overall findings of 43% antagonism, 31% synergism, and 26% additive, versus Crain et al. (2008) 38% antagonism, 36% synergism, and 26% additive based on an identical dataset. While this discrepancy is fairly modest at first glance, when coupled with the underlying conceptual issues outlined in our introduction, it highlights the need for a conceptual discussion in the ecological community regarding the interpretation of synergism and antagonism in situations where individual stressor effects oppose each other or where predicted cumulative effects are reversed and enhanced.

Our finding that the representation of interaction classes varied markedly by interaction type highlights the need to consider the direction of individual stressor effects when determining the occurrence of synergisms and antagonisms. This is particularly pertinent given the bias toward investigating and reporting adverse multiple stressor effects (i.e., double-negative interaction type) in ecology (e.g., reviews by Darling and Cote 2008; Ban et al. 2014; Klaminder et al. 2014). Based on our findings, double-positive interactions conformed to an additive effects model most frequently, but 63% of observed cases were nonadditive, and this proportion was larger for the other five interaction types. This further supports the notion that multiple stressors may interact to generate “ecological surprises” (e.g., Paine et al. 1998) more often than simply producing additive effects (Crain et al. 2008; Darling and Cote 2008).

### Contrasting concepts of synergisms and antagonisms and the need for a systematic approach

As an emerging field in ecology, there are increasing numbers of multiple stressor studies reporting synergisms and antagonisms that are based on imprecise descriptions or simply the judgment of the authors (Dunne 2010). These inconsistencies are aggravated by recent reviews and meta-analyses that define and measure these interactions differently and, therefore, are not comparative studies of any exact phenomenon (Vanhoudt et al. 2012). For example, Holmstrup et al. (2010) defined synergism and antagonism as “a convenient way to indicate combinations of a set of stressors that result in greater or lesser effects than expected from the single exposures” in their evaluation of 150 studies of stressors including heat, cold, desiccation, oxygen depletion, pathogens, and immunomodulatory factors combined with a variety of environmental pollutants. In contrast, Darling and Cote (2008) applied an additive null model in their evaluation of 112 multiple stressor experiments on animal mortality in freshwater, marine, and terrestrial communities. Not surprisingly, Holmstrup et al. (2010) reported synergisms in over half of their studies, whereas Darling and Cote (2008) reported synergisms in only a third, most likely reflecting the upward bias of Holmstrup et al.'s (2010) definition of synergy versus Darling and Cote's (2008) downward bias (a weakness of an additive effects model when individual effects are large or numerous; Folt et al. 1999). Acknowledging these differences of interpretation, Vanhoudt et al. (2012) applied their own definitions of synergism and antagonism based on “Concentration Addition” or “Independent Action” toxicological models. Due to the lack of support for either of these models, the authors then devised their own simplifying terminology to give an overview of their results as “positive interactions” (describing additive, synergism, “superaddition,” “potentiation,” or “increased effects”), “negative interactions” (loosely, antagonism), or no interactions. Interestingly, Vanhoudt et al. (2012) strongly opposed additive effect summation as an erroneous principle for determining synergism or antagonism despite this being the linear null model assumed in prevailing statistical techniques such as ANOVA and in our directional classification approach (Folt et al. 1999). Their explanation for this opposition is the fact that additive effect summation may result in cumulative effect estimates that exceed 100% (a particular problem when estimating standardized rate responses such as mortality where exceeding 100% is impossible). While multiplicative null models may correct for these “overestimates” (cumulative effect equals the product of individual stressor effects; Darling et al. 2010), the mechanistic and statistical basis for such approaches remains controversial (Greco et al. 1996; Kendler and Gardner 2010). Moreover, in a multiplicative model, one cannot know what the effect of any single stressor is on the cumulative effect without knowing the fractional contribution of all other stressors in the model, even in the absence of the product terms (Kendler and Gardner 2010). Alternative approaches for inferring synergism and antagonism utilizing isobolographic analysis exist in the fields of pharmacology, toxicology, and pathology (Nelson and Kursar 1999). While isobolographic analysis presents some key advantages over additive and multiplicative models (see Dunne 2010), it seems of limited utility in many ecological contexts because it relies on establishing multiple levels of dose–concentration relationships (a substantial ecological challenge in itself) and is further limited by implicitly assuming synergy only when effects are negative, reflecting its toxicological origins. Consequently, if the aim of identifying synergisms or antagonisms among multiple stressors is to determine whether ecological responses can be predicted from knowledge of single stressor effects, we encourage the use of an additive effects model (Kendler and Gardner 2010).

### Wider applicability of the classification system

Distinguishing classes of potential interactions between multiple stressors is important for elucidating stressor mechanisms and for separating effects based on severity (Folt et al. 1999). Irrespective of the experimental design, it is possible to reduce any multilevel or multivariable relationship to a set of two-variable relationships, but if higher-order interactions or highly influential covariates exist, it is possible that the effect direction can change from positive to negative (Nakagawa and Cuthill 2007). We have conceptualized a systematic approach for distinguishing such instances based on an additive effects model. While our examples only demonstrate two stressor interactions, the approach may also be applied when more than two stressors are operating, note that synergisms may be more likely to occur in such instances (Crain et al. 2008). The approach is potentially adaptable for testing against a multiplicative model (i.e., log additive; Folt et al. 1999). However, in this case, we recommend following the advice of Folt et al. (1999) that nonadditive responses should not be called synergisms or antagonisms so as to avoid confusion with the additive model.

A conceptually robust definition and systematic classification of synergism and antagonism is a prerequisite for improving our ability to predict and manage the interactive effects of multiple stressors. We have illustrated how the typical direction-independent classification of these terms may prove problematic and have conceptualized a directionally oriented extension of the traditional framework. Although some researchers may view the presence of interaction classes and directional interaction types as unduly complicated, the incorporation of these categories overcomes the limitations of the traditional framework when confronted with the more complex outcomes that appear to be quite commonplace. Moreover, the complexity of the framework translates into more informative descriptions and straightforward interpretations of complex interactions, which would otherwise be difficult even to describe.

### Management implications

It is generally assumed that multiple stressor synergies represent a “worst-case” scenario for ecosystem management (Paine et al. 1998; Folt et al. 1999). Consequently, our finding that antagonisms are generally more common than synergisms might be perceived as a “better case” scenario for managers. However, this is a misguided assumption because any nonadditive interaction, regardless of whether it is synergistic or antagonistic, poses complex challenges to ecosystem managers. This is especially true in mitigation or restoration situations where multiple stressors are already in operation and where impact and recovery trajectories are aligned (Scheffer et al. 2001). Here, it can be argued that antagonisms represent a particularly unfortunate scenario because efforts to reduce or eliminate a stressor may not yield proportional benefits unless a dominant stressor is driving the interaction (Halpern et al. 2008; Brown et al. 2013). On the other hand, synergisms can represent an optimistic scenario in certain situations (excluding mitigating synergisms), because efforts to reduce any particular stressor may yield larger overall benefits than otherwise expected (Crain et al. 2008). In contrast, mitigating synergisms suggest that the modes of action of single stressor effects are either eliminated when the stressors are combined or they surpass a critical threshold, such that the mechanism of the cumulative effect is opposite to that of the single stressor effects. Finally, additive effects suggest that stressors are operating independently of each other, so mitigation of any of the individual stressors will yield predictable benefits (Darling and Cote 2008). An ongoing challenge is to determine which stressors interact to generate nonadditive effects and to disentangle the mechanistic pathways by which multiple stressors interact in ecosystems.

## Supporting Information

**Figure S1.** Percentages of interaction classes by interaction type summarizing 170 studies manipulating two or more stressors in marine and coastal systems; reclassified from Crain et al. (8).

**Table S1.** Comprehensive listing of studies used for analysis of cumulative effects of stressor pairs.
