# Supplementary material for: Reconceptualizing synergism and antagonism among multiple stressors
Source: Ecol Evol. 2015 Mar 11;5(7):1538–47. doi: 10.1002/ece3.1465 (PMC4395182; doi:10.1002/ece3.1465)

## Double negative (n=70)

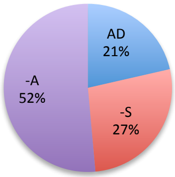


## Double positive (n=38)

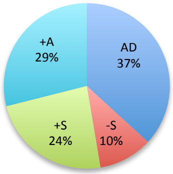


## Opposing (n=49)

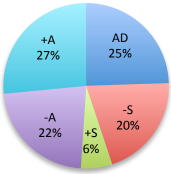


## Negative Neutral (n=9)

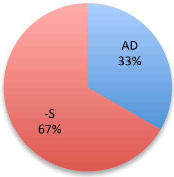


## Positive Neutral (n=3)

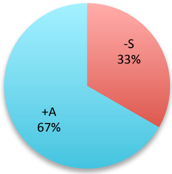


## Double Neutral (n=1)

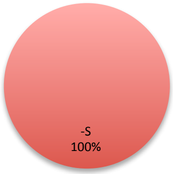


## Total (n=170)

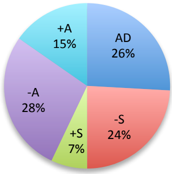


## Total w/o direction (n=170)

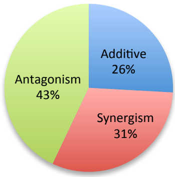

Supplement: Supplementary file 1 — Figure S1. Percentages of interaction classes by interaction type summarizing 170 studies manipulating two or more stressors in marine and coastal systems; reclassified from Crain et al. (8). [file ece30005-1538-sd1.pdf]
