# Supplementary material for: Reconceptualizing synergism and antagonism among multiple stressors
Source: Ecol Evol. 2015 Mar 11;5(7):1538–47. doi: 10.1002/ece3.1465 (PMC4395182; doi:10.1002/ece3.1465)
Supplement: Supplementary file 2 — Table S1. Comprehensive listing of studies used for analysis of cumulative effects of stressor pairs. [file ece30005-1538-sd2.doc]

Table S1. Comprehensive listing of studies used for analysis of cumulative effects of stressor pairs. Listed are stressor pairs, response level (p: population, c: community), habitat, taxon group, species information (for species-level studies), specific response level**,** individual effect of each stressor (Stress A and B compared to control), interaction effect (CI; confidence interval value), 95% confidence intervals for CI, additive predicted effect, interaction classification according to Crain *et al.* (2008), interaction type and, finally, directional interaction classification. The Table is modified from Table S1 in Crain *et al.* (2008), reordered by interaction type.

| Study | Stress A | Stress B | Response Level | Habitat | Taxa group | *Species* | Response variable | Individual Effect Stress A | Individual Effect Stress B | Interaction Effect | CI value | Interaction upper CI | Interaction lower CI | Additive Effect | Crain *et al.* (2008) Classification | Interaction Type | Directional Classification |
| --- | --- | --- | --- | --- | --- | --- | --- | --- | --- | --- | --- | --- | --- | --- | --- | --- | --- |
| 1 | CO2 | disease | p | rocky intertidal | invertebrate | *Mytilus edulis* | survival | -0.54 | -7.02 | -0.47 | -1.39 | 0.92 | -1.86 | -7.56 | AD | -- | AD |
| 2 | CO2 | nutrient | p | coral reef | invertebrate | *Porites compressa* | growth rate | -0.75 | -2.27 | 0.35 | -0.2 | 0.55 | 0.14 | -3.02 | A | -- | -A |
| 3 | CO2 | sediment | p | coral reef | invertebrate | *Porites compressa* | growth rate | -2.36 | -5.54 | 1.87 | -0.22 | 2.09 | 1.66 | -7.9 | A | -- | -A |
| 8 | hypoxia | disturb | p | oyster reef | invertebrate | *Crassostrea virginica* | survival | -0.21 | -0.29 | -12.3 | -0.86 | -11.44 | -13.16 | -0.5 | S | -- | -S |
| 9 | nutrient | CO2 | p | coral reef | invertebrate | *Acropora cervicornis* | growth rate | -4.33 | -4.25 | 4.98 | -1.85 | 6.83 | 3.13 | -8.58 | A | -- | -A |
| 15 | nutrient | temp | p | coral reef | invertebrate | *Porites cylindrica* | biomass | -0.09 | -0.44 | 0.27 | -0.14 | 0.41 | 0.12 | -0.53 | A | -- | -A |
| 17 | nutrient | temp | c | estuary | phytoplankton | *NA* | biomass | -0.46 | -0.59 | 1.59 | -0.59 | 2.18 | 1 | -1.05 | A | -- | -A |
| 17 | nutrient | toxin | c | sediment | invertebrate | *NA* | abundance | -3.57 | -4.43 | 3.68 | -0.31 | 3.99 | 3.36 | -8 | A | -- | -A |
| 19 | nutrient | toxin | c | estuary | invertebrate | *NA* | growth rate | -1.37 | -1 | 1.84 | -0.46 | 2.3 | 1.38 | -2.37 | A | -- | -A |
| 19 | nutrient | toxin | c | estuary | invertebrate | *NA* | growth rate | -0.35 | -0.19 | 0.63 | -0.44 | 1.07 | 0.19 | -0.54 | A | -- | -A |
| 20 | nutrient | toxin | c | sediment | invertebrate | *NA* | biomass | -1.87 | -1.71 | 2.28 | -0.87 | 3.15 | 1.42 | -3.58 | A | -- | -A |
| 25 | salinity | CO2 | p | seagrass | macrophyte | *Halophila johnsonii* | productivity | -1.28 | -1.53 | 1.45 | -0.27 | 1.72 | 1.18 | -2.81 | A | -- | -A |
| 1 | salinity | disease | p | rocky intertidal | invertebrate | *Mytilus edulis* | survival | -1.29 | -13.58 | -3.17 | -1.59 | -1.58 | -4.76 | -14.87 | S | -- | -S |
| 32 | salinity | SLR | p | salt marsh | macrophyte | *Spartina alterniflora* | biomass | -0.4 | -0.4 | 0.43 | -0.44 | 0.87 | -0.01 | -0.8 | AD | -- | AD |
| 33 | salinity | SLR | c | oligohaline marsh | macrophyte | *NA* | biomass | -1.04 | -0.17 | -1.19 | -0.58 | -0.61 | -1.76 | -1.21 | S | -- | -S |
| 35 | salinity | SLR | p | salt marsh | macrophyte | *Distichlis spicata* | biomass | -0.76 | -0.38 | 0.21 | -0.13 | 0.34 | 0.07 | -1.14 | A | -- | -A |
| 36 | salinity | SLR | p | salt marsh | macrophyte | *Aster* | biomass | -6.39 | -3.97 | 4.16 | -0.45 | 4.61 | 3.72 | -10.36 | A | -- | -A |
| 36 | salinity | SLR | p | salt marsh | fungi | *NA* | abundance | -10.03 | -3.34 | 3.49 | -0.42 | 3.91 | 3.07 | -13.37 | A | -- | -A |
| 37 | salinity | SLR | p | salt marsh | macrophyte | *Juncus ferardi* | biomass | -0.96 | -1.18 | 1.2 | -0.82 | 2.02 | 0.38 | -2.14 | A | -- | -A |
| 23 | salinity | SLR | p | marsh | tree | *Taxodium distichum* | biomass | -0.74 | -0.92 | 0.62 | -0.57 | 1.19 | 0.05 | -1.66 | A | -- | -A |
| 23 | salinity | SLR | p | marsh | tree | *Nyssa aquatica* | biomass | -1 | -0.18 | -0.51 | -0.57 | 0.06 | -1.08 | -1.18 | AD | -- | AD |
| 39 | salinity | SLR | p | estuary | macrophyte | *Juncus krausii* | biomass | -0.09 | -2.89 | 0 | -0.57 | 0.57 | -0.57 | -2.98 | AD | -- | AD |
| 40 | salinity | temp | p | seagrass | macrophyte | *Thalassia testudinum* | biomass | -1.82 | -0.17 | -1.27 | -0.82 | -0.45 | -2.09 | -1.99 | S | -- | -S |
| 41 | salinity | temp | p | zooplankton | invertebrate | *Brachionus rotundiformis* | abundance | -7.57 | -8.77 | 9.22 | -1.53 | 10.75 | 7.69 | -16.34 | A | -- | -A |
| 42 | salinity | temp | p | rocky intertidal | invertebrate | *Bembicium nanum* | survival | -0.7 | -0.13 | -0.66 | -0.36 | -0.3 | -1.02 | -0.83 | S | -- | -S |
| 43 | salinity | temp | p | sediment | invertebrate | *Spirorbis spirorbis* | survival | -0.18 | -0.67 | -0.36 | -0.2 | -0.16 | -0.57 | -0.85 | S | -- | -S |
| 43 | salinity | temp | p | sediment | invertebrate | *Circeus spirillum* | survival | -4.73 | -5.94 | 5.49 | -0.29 | 5.78 | 5.21 | -10.67 | A | -- | -A |
| 44 | salinity | temp | p | fouling community | invertebrate | *Hydroides elegans* | survival | -2.41 | -0.1 | -0.22 | -0.8 | 0.58 | -1.02 | -2.51 | AD | -- | AD |
| 48 | salinity | toxin | p | coral reef | invertebrate | *Porites lutea* | productivity | -0.72 | -0.8 | 0.56 | -0.23 | 0.79 | 0.33 | -1.52 | A | -- | -A |
| 41 | salinity | toxin | p | seagrass | macrophyte | *Thalassia testudinum* | biomass | -0.48 | -0.29 | 0.06 | -0.8 | 0.86 | -0.74 | -0.77 | AD | -- | AD |
| 41 | salinity | toxin | p | zooplankton | invertebrate | *Brachionus rotundiformis* | abundance | -8.26 | -17.71 | 7.73 | -1.36 | 9.09 | 6.38 | -25.97 | A | -- | -A |
| 49 | salinity | toxin | p | coral reef | macroalgae | *Enteromorpha intestinalis* | productivity | -3.63 | -1.02 | 1.13 | -0.45 | 1.58 | 0.68 | -4.65 | A | -- | -A |
| 46 | salinity | toxin | p | tidepool | invertebrate | *Tigripous japonicus* | survival | -0.22 | -5.63 | 3.42 | -0.66 | 4.08 | 2.76 | -5.85 | A | -- | -A |
| 51 | salinity | UV | p | subtidal | macroalgae | *Devaleraea ramentacea* | productivity | -2.36 | -1.74 | 1.27 | -0.21 | 1.48 | 1.06 | -4.1 | A | -- | -A |
| 51 | salinity | UV | p | subtidal | macroalgae | *Palmaria palmata* | productivity | -2.16 | -1.48 | 1.86 | -0.22 | 2.08 | 1.64 | -3.64 | A | -- | -A |
| 42 | salinity | UV | p | rocky intertidal | invertebrate | *Bembicium nanum* | survival | -0.01 | -0.16 | -1.13 | -0.36 | -0.77 | -1.5 | -0.17 | S | -- | -S |
| 52 | salinity | nutrient | p | seagrass | macrophyte | *Thalassia testudinum* | survival | -4.75 | -0.12 | -0.12 | -0.21 | 0.09 | -0.33 | -4.87 | AD | -- | AD |
| 53 | salinity | temp | p | coral reef | invertebrate | *Montastrea annularis* | productivity | -1.28 | -5.98 | 4.01 | -0.44 | 4.45 | 3.57 | -7.26 | A | -- | -A |
| 54 | sediment | invasive | c | rocky subtidal | macroalgae | *NA* | abundance | -12.34 | -10.91 | 7.86 | -0.61 | 8.47 | 7.24 | -23.25 | A | -- | -A |
| 60 | temp | disease | p | coral reef | invertebrate | *Gorgonia ventalina* | abundance | -5.01 | -1.56 | -2.4 | -0.25 | -2.15 | -2.65 | -6.57 | S | -- | -S |
| 63 | temp | fishing | p | n/a | invertebrate | *Brachionus plicatilis* | abundance | -1.06 | -0.79 | -1.55 | -0.59 | -0.96 | -2.13 | -1.85 | S | -- | -S |
| 65 | temp | hypoxia | p | estuary | invertebrate | *Perna perna* | survival | -0.92 | -0.69 | 0.13 | -0.04 | 0.17 | 0.09 | -1.61 | A | -- | -A |
| 66 | temp | nutrient | p | kelp forest | invertebrate | *Haliotis bermudense* | growth rate | -4.03 | -2.43 | 1.57 | -0.83 | 2.4 | 0.74 | -6.46 | A | -- | -A |
| 41 | temp | toxin | p | seagrass | macrophyte | *Thalassia testudinum* | biomass | -0.04 | -0.26 | -0.19 | -0.8 | 0.61 | -0.99 | -0.3 | AD | -- | AD |
| 41 | temp | toxin | p | zooplankton | invertebrate | *Brachionus rotundiformis* | abundance | -9.25 | -17.13 | 8.9 | -1.49 | 10.39 | 7.41 | -26.38 | A | -- | -A |
| 68 | temp | toxin | p | coral reef | invertebrate | *Porites cylindrica* | productivity | -0.47 | -0.37 | 0.32 | -0.21 | 0.53 | 0.12 | -0.84 | A | -- | -A |
| 42 | temp | UV | p | rocky intertidal | invertebrate | *Dolabrifera brazieri* | survival | -0.04 | -1.54 | 0.72 | -0.36 | 1.08 | 0.36 | -1.58 | A | -- | -A |
| 42 | temp | UV | p | rocky intertidal | invertebrate | *Bembicium nanum* | survival | -1.01 | -0.19 | 0.81 | -0.36 | 1.17 | 0.45 | -1.2 | A | -- | -A |
| 69 | temp | UV | p | coral reef | invertebrate | *Eunicea tourneforti* | abundance | -0.66 | -0.07 | -0.81 | -0.05 | -0.76 | -0.87 | -0.73 | S | -- | -S |
| 71 | temp | UV | p | coral reef | phytoplankton | *Symbiodinium* | productivity | -10.16 | -0.46 | -6.78 | -1.25 | -5.53 | -8.03 | -10.62 | S | -- | -S |
| 74 | temp | UV | p | coral reef | invertebrate | *Stylophora pistillata* | productivity | -4.4 | -2.94 | -0.74 | -0.04 | -0.7 | -0.78 | -7.34 | S | -- | -S |
| 74 | temp | UV | p | coral reef | invertebrate | *Pavona cactus* | productivity | -0.65 | -3.58 | -8.16 | -0.07 | -8.09 | -8.23 | -4.23 | S | -- | -S |
| 74 | temp | UV | p | coral reef | invertebrate | *Acropora sp.* | productivity | -0.64 | -3.18 | -7.98 | -0.07 | -7.91 | -8.04 | -3.82 | S | -- | -S |
| 74 | temp | UV | p | coral reef | invertebrate | *Montipora aequituberculata* | productivity | -2.42 | -5.53 | 2.43 | -0.04 | 2.47 | 2.38 | -7.95 | A | -- | -A |
| 75 | temp | UV | p | coral reef | invertebrate | *Palythoa caribaeorum* | abundance | -0.99 | -0.5 | -0.18 | -0.43 | 0.25 | -0.62 | -1.49 | AD | -- | AD |
| 46 | toxin | temp | p | tidepool | invertebrate | *Tigripous japonicus* | survival | -12.7 | -0.87 | -4.12 | -0.7 | -3.42 | -4.82 | -13.57 | S | -- | -S |
| 46 | toxin | temp | p | tidepool | invertebrate | *Tigripous japonicus* | survival | -3.84 | -1.35 | 0.87 | -0.58 | 1.45 | 0.3 | -5.19 | A | -- | -A |
| 78 | toxin | UV | p | sediment | invertebrate | *Paramoera walkeri* | survival | -0.86 | -0.98 | -1.21 | -1.42 | 0.21 | -2.62 | -1.84 | AD | -- | AD |
| 79 | toxin | UV | c | estuary | phytoplankton | *plankton* | abundance | -11.98 | -9.02 | 9.38 | -1.55 | 10.93 | 7.83 | -21 | A | -- | -A |
| 80 | toxin | UV | c | estuary | phytoplankton | *plankton* | abundance | -27.38 | -16.64 | 18.38 | -2.72 | 21.1 | 15.66 | -44.02 | A | -- | -A |
| 82 | toxin | UV | p | estuary | invertebrate | *Panopeus herbstii* | survival | -0.02 | -0.07 | -0.94 | -1.41 | 0.47 | -2.34 | -0.09 | AD | -- | AD |
| 84 | toxin | UV | p | estuary | invertebrate | *Mysidopsis bahia* | survival | -184.19 | -44.8 | -55.51 | -1.33 | -54.18 | -56.84 | -228.99 | S | -- | -S |
| 84 | toxin | UV | p | estuary | invertebrate | *Mysidopsis bahia* | biomass | -6.69 | -2.07 | -1.4 | -0.14 | -1.26 | -1.54 | -8.76 | S | -- | -S |
| 86 | toxin | UV | p | estuary | vertebrate | *Menidia beryllina* | survival | -353.81 | -100.76 | -256.61 | -36.3 | -220.31 | -292.92 | -454.57 | S | -- | -S |
| 87 | toxin | UV | c | pelagic | phytoplankton | *NA* | abundance | -2.6 | -4.42 | 1.08 | -0.81 | 1.89 | 0.26 | -7.02 | A | -- | -A |
| 89 | toxin | UV | p | seagrass | bacteria | *Vibrio fischeri* | productivity | -0.64 | -7.87 | -0.56 | -0.8 | 0.24 | -1.36 | -8.51 | AD | -- | AD |
| 89 | toxin | UV | p | seagrass | bacteria | *Vibrio fischeri* | productivity | -6.63 | -4.36 | 0.2 | -0.8 | 1 | -0.6 | -10.99 | AD | -- | AD |
| 89 | toxin | UV | p | seagrass | bacteria | *Vibrio fischeri* | productivity | -8.2 | -2.79 | 0.2 | -0.8 | 1 | -0.6 | -10.99 | AD | -- | AD |
| 89 | toxin | UV | p | seagrass | invertebrate | *Lytechinus variegatus* | survival | -5.17 | -2.03 | -1.42 | -0.58 | -0.84 | -2 | -7.2 | S | -- | -S |
| 89 | toxin | UV | p | seagrass | invertebrate | *Lytechinus variegatus* | survival | -4.09 | -4 | 0.37 | -0.56 | 0.93 | -0.2 | -8.09 | AD | -- | AD |
| 20 | nutrient | toxin | c | sediment | n/a | *NA* | productivity | -0.17 | 0.17 | -0.38 | -0.8 | 0.42 | -1.18 | 0 | AD | -+ | AD |
| 28 | salinity | nutrient | p | estuary | macroalgae | *Enteromorpha intestinalis* | biomass | -2.21 | 0.88 | 1.09 | -0.44 | 1.53 | 0.64 | -1.33 | A | -+ | -A |
| 29 | salinity | nutrient | p | seagrass | macrophyte | *Zostera marina* | abundance | -0.3 | 0.39 | -0.83 | -1.4 | 0.57 | -2.23 | 0.09 | AD | -+ | AD |
| 29 | salinity | nutrient | p | seagrass | macrophyte | *Zostera marina* | abundance | -0.47 | 1.18 | -1.44 | -1.43 | -0.01 | -2.87 | 0.71 | AD | -+ | AD |
| 30 | salinity | nutrient | c | marsh | cyanobacteria | *NA* | productivity | -0.36 | 1.26 | -0.54 | -0.57 | 0.03 | -1.11 | 0.9 | AD | -+ | AD |
| 23 | salinity | nutrient | p | marsh | tree | *Taxodium distichum* | biomass | -0.33 | 1.31 | 0 | -0.57 | 0.57 | -0.57 | 0.98 | AD | -+ | AD |
| 23 | salinity | nutrient | p | marsh | tree | *Nyssa aquatica* | biomass | -0.48 | 2.81 | -2.51 | -0.62 | -1.89 | -3.13 | 2.33 | S | -+ | +A |
| 33 | salinity | SLR | c | oligohaline marsh | macrophyte | *NA* | biomass | -1.09 | 0.03 | -0.84 | -0.57 | -0.27 | -1.41 | -1.06 | S | -+ | -S |
| 35 | salinity | SLR | p | brackish marsh | macrophyte | *Schoenoplectus californicus* | biomass | -2.69 | 0.05 | 0.22 | -0.17 | 0.39 | 0.05 | -2.64 | A | -+ | -A |
| 38 | salinity | SLR | p | salt marsh | macrophyte | *Phragmites australis* | productivity | -2.13 | 2.93 | -4.11 | -0.98 | -3.13 | -5.1 | 0.8 | S | -+ | -S |
| 42 | salinity | temp | p | rocky intertidal | invertebrate | *Dolabrifera brazieri* | survival | -0.56 | 0.36 | -2.29 | -0.39 | -1.9 | -2.67 | -0.2 | S | -+ | -S |
| 49 | salinity | toxin | p | coral reef | macroalgae | *Gracilaria tenuistipitata* | productivity | -1.32 | 0.44 | -0.93 | -0.44 | -0.49 | -1.37 | -0.88 | S | -+ | -S |
| 61 | temp | disease | p | rocky subtidal | invertebrate | *Haliotis rufescens* | survival | -11 | 1.13 | -13.34 | -0.17 | -13.17 | -13.51 | -9.87 | S | -+ | -S |
| 70 | temp | UV | p | subtidal | macroalgae | *Ulvaclathrata* | productivity | -4.31 | 0.65 | 2.92 | -0.9 | 3.82 | 2.02 | -3.66 | A | -+ | -A |
| 79 | toxin | UV | c | estuary | bacteria | *bacteria* | abundance | -2.38 | 10.06 | -0.93 | -0.81 | -0.12 | -1.74 | 7.68 | S | -+ | +A |
| 85 | toxin | UV | p | estuary | invertebrate | *Mulinia lateralis* | survival | -4.78 | 0.28 | -0.32 | -0.8 | 0.48 | -1.12 | -4.5 | AD | -+ | AD |
| 91 | nutrient | fishing | c | coral reef | macroalgae | NA | biomass | -0.17 | 0.34 | 0.94 | -0.56 | 1.5 | 0.37 | 0.17 | A | -+ | +S |
| 7 | hypoxia | disturb | c | oyster reef | vertebrate | *NA* | abundance | -0.82 | 0 | -6.09 | -0.65 | -5.44 | -6.73 | -0.82 | S | -0 | -S |
| 82 | toxin | UV | p | estuary | invertebrate | *Menippe adina* | survival | -0.03 | 0 | -0.58 | -1.39 | 0.81 | -1.97 | -0.03 | AD | -0 | AD |
| 85 | toxin | UV | p | estuary | invertebrate | *Mysidopsis bahia* | survival | -3.55 | 0 | -0.29 | -0.8 | 0.51 | -1.09 | -3.55 | AD | -0 | AD |
| 85 | toxin | UV | p | estuary | invertebrate | *Mysidopsis bahia* | survival | -6.4 | 0 | -1.81 | -0.84 | -0.97 | -2.65 | -6.4 | S | -0 | -S |
| 85 | toxin | UV | p | estuary | invertebrate | *Mulinia lateralis* | survival | -0.38 | 0 | -7.57 | -1.34 | -6.23 | -8.91 | -0.38 | S | -0 | -S |
| 85 | toxin | UV | p | estuary | invertebrate | *Mulinia lateralis* | survival | -1.34 | 0 | -2.31 | -0.87 | -1.44 | -3.17 | -1.34 | S | -0 | -S |
| 85 | toxin | UV | p | estuary | invertebrate | *Mulinia lateralis* | survival | -0.39 | 0 | -0.5 | -0.8 | 0.3 | -1.31 | -0.39 | AD | -0 | AD |
| 4 | CO2 | UV | p | salt marsh | macrophyte | *Elymus athericus* | biomass | 0.5 | -0.25 | -0.33 | -0.15 | -0.18 | -0.47 | 0.25 | S | +- | +A |
| 7 | hypoxia | disturb | c | oyster reef | invertebrate | *NA* | abundance | 0.65 | -0.38 | -2.89 | -0.49 | -2.4 | -3.39 | 0.27 | S | +- | -S |
| 10 | nutrient | disease | p | coral reef | invertebrate | *Monastrea sp.* | disease severity | 0.11 | -0.15 | -1.21 | 0.22 | -1.43 | -1 | -0.04 | S | +- | -S |
| 12 | nutrient | SLR | p | marsh | tree | *Salix nigra* | biomass | 3.21 | -0.09 | -2.64 | -0.19 | -2.45 | -2.82 | 3.12 | S | +- | +A |
| 17 | nutrient | toxin | c | sediment | invertebrate | *NA* | abundance | 1.57 | -1.46 | -0.87 | -0.27 | -0.6 | -1.13 | 0.11 | S | +- | +A |
| 18 | nutrient | toxin | c | sediment | invertebrate | *NA* | productivity | 0.07 | -0.34 | 0.14 | -0.21 | 0.35 | -0.07 | -0.27 | AD | +- | AD |
| 19 | nutrient | toxin | c | estuary | phytoplankton | *NA* | productivity | 2.86 | -0.12 | -1.93 | -0.47 | -1.46 | -2.39 | 2.74 | S | +- | +A |
| 19 | nutrient | toxin | c | estuary | phytoplankton | *NA* | abundance | 1.19 | -0.34 | -0.89 | -0.44 | -0.45 | -1.34 | 0.85 | S | +- | +A |
| 19 | nutrient | toxin | c | estuary | invertebrate | *NA* | abundance | 0.52 | -0.09 | -0.4 | -0.44 | 0.04 | -0.84 | 0.43 | AD | +- | AD |
| 20 | nutrient | toxin | c | sediment | bacteria | *NA* | abundance | 0.83 | -0.09 | 1.71 | -0.84 | 2.55 | 0.88 | 0.74 | A | +- | +S |
| 21 | nutrient | UV | c | phytoplankton | phytoplankton | *NA* | productivity | 0.23 | -2.97 | 0.26 | -0.8 | 1.06 | -0.54 | -2.74 | AD | +- | AD |
| 22 | nutrient | UV | c | sediment | macroalgae | *NA* | biomass | 1.21 | -0.6 | 1.71 | -0.83 | 2.54 | 0.87 | 0.61 | A | +- | +S |
| 23 | nutrient | SLR | p | marsh | tree | *Taxodium distichum* | biomass | 1.5 | -0.46 | -0.65 | -0.57 | -0.08 | -1.22 | 1.04 | S | +- | +A |
| 23 | nutrient | SLR | p | marsh | tree | *Nyssa aquatica* | biomass | 2.51 | -0.08 | -1.11 | -0.58 | -0.53 | -1.68 | 2.43 | S | +- | +A |
| 24 | nutrient | SLR | p | salt marsh | macrophyte | *Elmyus pycnanthus* | biomass | 1.83 | -0.4 | -1.06 | -0.23 | -0.83 | -1.3 | 1.43 | S | +- | +A |
| 31 | salinity | nutrient | p | estuarine | macroalgae | *Enteromorpha spp.* | biomass | 1.08 | -0.2 | -0.95 | -0.57 | -0.38 | -1.53 | 0.88 | S | +- | +A |
| 34 | salinity | SLR | p | salt marsh | macrophyte | *Saggitaria lancifolia* | biomass | 4.71 | -1.07 | -4.74 | -0.57 | -4.17 | -5.31 | 3.64 | S | +- | +A |
| 46 | salinity | toxin | p | tidepool | invertebrate | *Tigripous japonicus* | survival | 2.52 | -1.51 | 0.78 | -0.57 | 1.35 | 0.21 | 1.01 | A | +- | -A |
| 42 | salinity | UV | p | rocky intertidal | invertebrate | *Dolabrifera brazieri* | survival | 0.26 | -1.85 | -1.56 | -0.37 | -1.19 | -1.93 | -1.59 | S | +- | -S |
| 42 | salinity | UV | p | rocky intertidal | invertebrate | *Siphonaria denticulata* | survival | 0.03 | -1.06 | 0.1 | -0.36 | 0.46 | -0.26 | -1.03 | AD | +- | AD |
| 42 | temp | UV | p | rocky intertidal | invertebrate | *Siphonaria denticulata* | survival | 0.04 | -1.5 | 1.1 | -0.37 | 1.47 | 0.74 | -1.46 | A | +- | -A |
| 70 | temp | UV | p | subtidal | macroalgae | *Ulva bulbosa* | productivity | 1.11 | -10.58 | 7.86 | -1.37 | 9.23 | 6.49 | -9.47 | A | +- | -A |
| 72 | temp | UV | p | rocky intertidal | macroalgae | *Alaria marginata* | abundance | 0.91 | -7.27 | 4.26 | -0.45 | 4.71 | 3.81 | -6.36 | A | +- | -A |
| 72 | temp | UV | p | rocky intertidal | macroalgae | *Fucus gardneri* | abundance | 3.66 | -1.63 | 0.42 | -0.36 | 0.78 | 0.06 | 2.03 | A | +- | -A |
| 73 | temp | UV | p | rocky intertidal | macroalgae | *Fucus spiralis* | growth rate | 10.5 | -7.69 | 2.02 | -0.85 | 2.87 | 1.17 | 2.81 | A | +- | -A |
| 73 | temp | UV | p | rocky intertidal | macroalgae | *Fucus vesiculosus* | growth rate | 16.99 | -3.33 | -11.28 | -1.78 | -9.5 | -13.07 | 13.66 | S | +- | +A |
| 73 | temp | UV | p | rocky intertidal | macroalgae | *Fucus serratus* | growth rate | 4.9 | -2.62 | 1.29 | -0.82 | 2.11 | 0.47 | 2.28 | A | +- | -A |
| 82 | toxin | UV | p | estuary | invertebrate | *Callinectes sapidus* | survival | 0.03 | -0.02 | -0.81 | -1.4 | 0.59 | -2.21 | 0.01 | AD | +- | AD |
| 82 | toxin | UV | p | estuary | invertebrate | *Libinia dubia* | survival | 0.02 | -0.02 | -1.11 | -1.41 | 0.3 | -2.52 | 0 | AD | +- | AD |
| 87 | toxin | UV | c | pelagic | bacteria | *NA* | abundance | 0.95 | -1.91 | -4.85 | -1.06 | -3.79 | -5.9 | -0.96 | S | +- | -S |
| 89 | toxin | UV | p | seagrass | bacteria | *Vibrio fischeri* | productivity | 1.46 | -3.85 | 1.2 | -0.82 | 2.02 | 0.38 | -2.39 | A | +- | -A |
| 90 | nutrient | fishing | p | coral reef | invertebrate | *Oculina arbuscula* | growth rate | 0.42 | -0.14 | -0.73 | -0.15 | -0.58 | -0.87 | 0.28 | S | +- | -S |
| 5 | CO2 | UV | p | rocky subtidal | macroalgae | *Saccharina latissima* | biomass | 0.39 | 0.51 | -1.25 | -0.04 | -1.21 | -1.29 | 0.9 | S | ++ | **-S** |
| 5 | CO2 | UV | p | rocky subtidal | macroalgae | *Nereocystis luetkeana* | biomass | 0.41 | 0.7 | 0.53 | -0.04 | 0.57 | 0.49 | 1.11 | S | ++ | +S |
| 12 | nutrient | SLR | p | marsh | tree | *Taxodium distichum* | biomass | 2.27 | 0.32 | -0.93 | -0.18 | -0.75 | -1.1 | 2.59 | A | ++ | +A |
| 13 | nutrient | sediment | p | estuary | macrophyte | *Avicennia marina* | growth rate | 0.95 | 1.9 | -1.78 | -0.38 | -1.4 | -2.15 | 2.85 | A | ++ | +A |
| 13 | nutrient | sediment | p | estuary | macrophyte | *Avicennia marina* | growth rate | 0.23 | 1.09 | 0.73 | -0.36 | 1.09 | 0.37 | 1.32 | S | ++ | +S |
| 13 | nutrient | sediment | c | estuary | microorganisms | *NA* | productivity | 0.15 | 0.83 | -0.41 | -0.36 | -0.05 | -0.77 | 0.98 | AD | ++ | AD |
| 13 | nutrient | sediment | c | estuary | microorganisms | *NA* | productivity | 0.07 | 0.43 | 0.37 | -0.36 | 0.73 | 0.01 | 0.5 | S | ++ | +S |
| 14 | nutrient | temp | p | coral reef | invertebrate | *Pocillopora damicornis* | biomass | 0.58 | 0.17 | 0.49 | -0.44 | 0.93 | 0.05 | 0.75 | S | ++ | +S |
| 14 | nutrient | temp | p | coral reef | invertebrate | *Porites lobata* | biomass | 1.05 | 0.78 | 0.18 | -0.44 | 0.62 | -0.25 | 1.83 | AD | ++ | AD |
| 16 | nutrient | temp | p | rocky intertidal | macroalgae | *Enteromorpha intestinalis* | abundance | 0.12 | 0.67 | 0 | -0.57 | 0.57 | -0.57 | 0.79 | AD | ++ | AD |
| 17 | nutrient | temp | c | estuary | phytoplankton | *NA* | biomass | 0.3 | 0.2 | 0.08 | -0.57 | 0.65 | -0.48 | 0.5 | AD | ++ | AD |
| 17 | nutrient | toxin | c | sediment | invertebrate | *NA* | abundance | 5.27 | 0.18 | -3.66 | -0.31 | -3.35 | -3.97 | 5.45 | A | ++ | +A |
| 19 | nutrient | toxin | c | estuary | phytoplankton | *NA* | abundance | 0.06 | 0.07 | 1.6 | -0.46 | 2.06 | 1.15 | 0.13 | S | ++ | +S |
| 19 | nutrient | toxin | c | estuary | plankton | *NA* | abundance | 1.33 | 0.06 | -2.36 | -0.48 | -1.88 | -2.83 | 1.39 | A | ++ | **-S** |
| 19 | nutrient | toxin | c | estuary | vertebrate | *NA* | growth rate | 0.06 | 0.01 | -0.02 | -0.44 | 0.42 | -0.46 | 0.07 | AD | ++ | AD |
| 20 | nutrient | toxin | c | sediment | microalgae | *NA* | biomass | 2.39 | 0.43 | -1.25 | -0.82 | -0.43 | -2.07 | 2.82 | A | ++ | +A |
| 21 | nutrient | UV | c | phytoplankton | phytoplankton | *NA* | productivity | 1.25 | 1.25 | -0.2 | -0.8 | 0.6 | -1 | 2.5 | AD | ++ | AD |
| 21 | nutrient | UV | c | phytoplankton | phytoplankton | *NA* | productivity | 0.59 | 2.93 | -1.54 | -0.82 | -0.72 | -2.37 | 3.52 | A | ++ | +A |
| 16 | nutrient | UV | p | rocky intertidal | macroalgae | *Enteromorpha intestinalis* | abundance | 0.09 | 0.38 | 0.72 | -0.57 | 1.29 | 0.14 | 0.47 | S | ++ | +S |
| 24 | nutrient | SLR | p | salt marsh | macrophyte | *Puccinellia maritima* | biomass | 1 | 0.2 | -0.12 | -0.23 | 0.11 | -0.35 | 1.2 | AD | ++ | AD |
| 24 | nutrient | SLR | p | salt marsh | macrophyte | *Spartina angelica* | biomass | 1.85 | 0.28 | 0.58 | -0.23 | 0.81 | 0.34 | 2.13 | S | ++ | +S |
| 31 | salinity | nutrient | p | estuarine | macroalgae | *Enteromorpha spp.* | biomass | 0.73 | 0.18 | -0.14 | -0.57 | 0.43 | -0.71 | 0.91 | AD | ++ | AD |
| 32 | salinity | SLR | p | salt marsh | macrophyte | *Saggitaria lancifolia* | biomass | 0.24 | 3.23 | 0.26 | -0.43 | 0.69 | -0.18 | 3.47 | AD | ++ | AD |
| 24 | salinity | temp | p | seagrass | macrophyte | *Halophila johnsonii* | productivity | 0.15 | 0.59 | -0.51 | -0.26 | -0.25 | -0.77 | 0.74 | A | ++ | +A |
| 42 | salinity | temp | p | rocky intertidal | invertebrate | *Siphonaria denticulata* | survival | 0.1 | 0.6 | -1.34 | -0.37 | -0.97 | -1.71 | 0.7 | A | ++ | **-S** |
| 45 | salinity | temp | p | subtidal | phytoplankton | *Gyrodinium aureolum* | growth rate | 3.84 | 2.1 | 19.17 | -2.83 | 22 | 16.34 | 5.94 | S | ++ | +S |
| 49 | salinity | toxin | p | coral reef | invertebrate | *Trochus maculatus* | productivity | 1.04 | 0.54 | -1.01 | -0.13 | -0.88 | -1.15 | 1.58 | A | ++ | +A |
| 55 | sediment | nutrient | c | rocky intertidal | macroalgae | *NA* | abundance | 1.16 | 0.58 | 1.53 | -0.45 | 1.98 | 1.07 | 1.74 | S | ++ | +S |
| 57 | temp | CO2 | p | coral reef | invertebrate | *Stylophora pistillata* | abundance | 1.66 | 6.64 | -2.81 | -0.89 | -1.92 | -3.7 | 8.3 | A | ++ | +A |
| 58 | temp | CO2 | p | phytoplankton | cyanobacteria | *Prochlorococcus sp.* | growth rate | 0.33 | 0.33 | -1.13 | -0.82 | -0.31 | -1.95 | 0.66 | S | ++ | +A |
| 58 | temp | CO2 | p | phytoplankton | cyanobacteria | *Synechococcus sp.* | growth rate | 5.94 | 1.75 | -0.59 | -0.8 | 0.21 | -1.4 | 7.69 | AD | ++ | AD |
| 64 | temp | hypoxia | p | sediment | invertebrate | *Chorus giganteus* | biomass | 2.02 | 0.96 | -3.12 | -0.92 | -2.2 | -4.03 | 2.98 | A | ++ | +A |
| 16 | temp | UV | p | rocky intertidal | macroalgae | *Enteromorpha intestinalis* | abundance | 0.67 | 0.07 | 0.44 | -0.57 | 1.01 | -0.13 | 0.74 | AD | ++ | AD |
| 82 | toxin | UV | p | estuary | invertebrate | *Callinectes sapidus* | survival | 0.1 | 0.1 | -1.27 | -1.42 | 0.15 | -2.69 | 0.2 | AD | ++ | AD |
| 82 | toxin | UV | p | estuary | invertebrate | *Libinia dubia* | survival | 0.02 | 0.02 | -1.11 | -1.42 | 0.31 | -2.52 | 0.04 | AD | ++ | AD |
| 82 | toxin | UV | p | estuary | invertebrate | *Panopeus herbstii* | survival | 0.07 | 0.09 | -1.03 | -1.41 | 0.38 | -2.44 | 0.16 | AD | ++ | AD |
| 87 | toxin | UV | c | pelagic | bacteria | *NA* | abundance | 1.38 | 1.91 | -8.63 | -1.46 | -7.17 | -10.09 | 3.29 | A | ++ | **-S** |
| 92 | nutrient | fishing | c | coral reef | macroalgae | NA | abundance | 0.48 | 3.57 | -0.93 | -0.21 | -0.72 | -1.14 | 4.05 | A | ++ | +A |
| 88 | toxin | UV | p | estuary | phytoplankton | *Ankistrodesmus spp.* | abundance | 0 | 3 | -1.9 | -0.85 | -1.05 | -2.74 | 3 | A | 0+ | +A |
| 19 | nutrient | toxin | c | estuary | bacteria | *NA* | productivity | 4.47 | 0 | -2.83 | -0.49 | -2.34 | -3.32 | 4.47 | A | +0 | +A |
| 85 | toxin | UV | p | estuary | invertebrate | *Mulinia lateralis* | survival | 0.48 | 0 | -5.97 | -1.16 | -4.81 | -7.14 | 0.48 | S | +0 | **-S** |
| 46 | salinity | temp | p | tidepool | invertebrate | *Tigripous japonicus* | survival | 0 | -0.75 | -1.61 | -0.59 | -1.02 | -2.19 | -0.75 | S | 0- | -S |
| 61 | temp | disease | p | rocky subtidal | invertebrate | *Haliotis rufescens* | survival | 0 | -0.51 | -12.31 | -0.16 | -12.15 | -12.47 | -0.51 | S | 0- | -S |
| 85 | toxin | UV | p | estuary | invertebrate | *Mulinia lateralis* | survival | 0 | 0 | -4.19 | -0.99 | -3.2 | -5.19 | 0 | S | 00 | **-S** |
| 6 | disturb | SLR | c | oligohaline marsh | macrophyte | *NA* | species richness | NA | NA | NA | NA | NA | NA | NA | NA | NA | NA |
| 6 | disturb | SLR | c | oligohaline marsh | macrophyte | *NA* | species richness | NA | NA | NA | NA | NA | NA | NA | NA | NA | NA |
| 6 | disturb | SLR | c | oligohaline marsh | macrophyte | *NA* | species richness | NA | NA | NA | NA | NA | NA | NA | NA | NA | NA |
| 11 | nutrient | disease | p | coral reef | invertebrate | *Siderastrea siderea* | disease severity | NA | NA | NA | NA | NA | NA | NA | NA | NA | NA |
| 26 | salinity | CO2 | p | salt marsh | macrophyte | *Aster tripolium* | biomass | NA | NA | NA | NA | NA | NA | NA | NA | NA | NA |
| 26 | salinity | CO2 | p | salt marsh | macrophyte | *Puccinellia maritima* | biomass | NA | NA | NA | NA | NA | NA | NA | NA | NA | NA |
| 27 | salinity | CO2 | p | salt marsh | macrophyte | *Elymus athericus* | biomass | NA | NA | NA | NA | NA | NA | NA | NA | NA | NA |
| 27 | salinity | CO2 | p | salt marsh | macrophyte | *Spartina angelica* | biomass | NA | NA | NA | NA | NA | NA | NA | NA | NA | NA |
| 27 | salinity | SLR | p | salt marsh | macrophyte | *Spartina angelica* | biomass | NA | NA | NA | NA | NA | NA | NA | NA | NA | NA |
| 6 | salinity | SLR | c | oligohaline marsh | macrophyte | *NA* | species richness | NA | NA | NA | NA | NA | NA | NA | NA | NA | NA |
| 47 | salinity | temp | p | seagrass | macrophyte | *Zostera capricorni* | abundance | NA | NA | NA | NA | NA | NA | NA | NA | NA | NA |
| 50 | salinity | toxin | p | estuary | invertebrate | *Mysidopsis bahia* | survival | NA | NA | NA | NA | NA | NA | NA | NA | NA | NA |
| 27 | SLR | CO2 | p | salt marsh | macrophyte | *Spartina angelica* | biomass | NA | NA | NA | NA | NA | NA | NA | NA | NA | NA |
| 56 | temp | CO2 | p | salt marsh | macrophyte | *Spartina angelica* | biomass | NA | NA | NA | NA | NA | NA | NA | NA | NA | NA |
| 56 | temp | CO2 | p | salt marsh | macrophyte | *Puccinellia maritima* | biomass | NA | NA | NA | NA | NA | NA | NA | NA | NA | NA |
| 59 | temp | disease | p | coral reef | invertebrate | *Montastrea spp.* | survival | NA | NA | NA | NA | NA | NA | NA | NA | NA | NA |
| 1 | temp | disease | p | rocky intertidal | invertebrate | *Mytilus edulis* | survival | NA | NA | NA | NA | NA | NA | NA | NA | NA | NA |
| 62 | temp | disturb | p | coral reef | invertebrate | *Montastraea faveolata* | disease severity | NA | NA | NA | NA | NA | NA | NA | NA | NA | NA |
| 67 | temp | sediment | p | coral reef | invertebrate | *Acropora intermedia* | survival | NA | NA | NA | NA | NA | NA | NA | NA | NA | NA |
| 67 | temp | sediment | p | coral reef | invertebrate | *Acropora intermedia* | survival | NA | NA | NA | NA | NA | NA | NA | NA | NA | NA |
| 76 | toxin | hypoxia | p | oyster reef | invertebrate | *Crassostrea virginica* | survival | NA | NA | NA | NA | NA | NA | NA | NA | NA | NA |
| 77 | toxin | temp | p | seagrass | macrophyte | *Halodule wrightii* | biomass | NA | NA | NA | NA | NA | NA | NA | NA | NA | NA |
| 77 | toxin | temp | p | seagrass | macrophyte | *Thalassia testudinum* | biomass | NA | NA | NA | NA | NA | NA | NA | NA | NA | NA |
| 80 | toxin | UV | c | estuary | bacteria | *bacteria* | abundance | NA | NA | NA | NA | NA | NA | NA | NA | NA | NA |
| 81 | toxin | UV | p | rocky subtidal | invertebrate | *Paramoera walkeri* | survival | NA | NA | NA | NA | NA | NA | NA | NA | NA | NA |
| 83 | toxin | UV | p | coral reef | invertebrate | *Porites divaricata* | survival | NA | NA | NA | NA | NA | NA | NA | NA | NA | NA |
| 83 | toxin | UV | p | coral reef | invertebrate | *Porites divaricata* | survival | NA | NA | NA | NA | NA | NA | NA | NA | NA | NA |
| 83 | toxin | UV | p | coral reef | invertebrate | *Porites divaricata* | survival | NA | NA | NA | NA | NA | NA | NA | NA | NA | NA |
| 83 | toxin | UV | p | coral reef | invertebrate | *Porites divaricata* | survival | NA | NA | NA | NA | NA | NA | NA | NA | NA | NA |
| 85 | toxin | UV | p | estuary | invertebrate | *Mysidopsis bahia* | survival | NA | NA | NA | NA | NA | NA | NA | NA | NA | NA |
| 85 | toxin | UV | p | estuary | invertebrate | *Mysidopsis bahia* | survival | NA | NA | NA | NA | NA | NA | NA | NA | NA | NA |

Literature Cited

1. Babarro, J. M. F. & de Zwaan, A. (2002) Influence of abiotic factors on bacterial proliferation and anoxic survival of the sea mussel Mytilus edulis L. *J Exp Mar Biol Ecol* 273**:**33-49.

2. Marubini, F. & Atkinson, M. J. (1999) Effects of lowered pH and elevated nitrate on coral calcification. *Mar Ecol Prog Ser* 188**:**117-121.

3. Marubini, F., Barnett, H., Langdon, C. & Atkinson, M. J. (2001) Dependence of calcification on light and carbonate ion concentration for the hermatypic coral Porites compressa. *Mar Ecol Prog Ser* 220**:**153-162.

4. Van de Staaij, J. W. M., Lenssen, G. M., Stroetenga, M. & Rozema, J. (1993) The Combined Effects of Elevated Co2 Levels and Uv-B Radiation on Growth-Characteristics of Elymus-Athericus (= E-Pycnanathus). *Vegetatio* 104**:**433-439.

5. Swanson, A. K. & Fox, C. H. (2007) Altered kelp (Laminariales) phlorotannins and growth under elevated carbon dioxide and ultraviolet-B treatments can influence associated intertidal food webs. *Global Change Biol* 13**:**1696-1709.

6. Baldwin, A. H., McKee, K. L. & Mendelssohn, I. A. (1996) The influence of vegetation, salinity, and inundation on seed banks of oligohaline coastal marshes. *Am J Bot* 83**:**470-479.

7. Lenihan, H. S., Peterson, C. H., Byers, J. E., Grabowski, J. H., Thayer, G. W. & Colby, D. R. (2001) Cascading of habitat degradation: Oyster reefs invaded by refugee fishes escaping stress. *Ecol Appl* 11**:**764-782.

8. Lenihan, H. S. & Peterson, C. H. (1998) How habitat degradation through fishery disturbance enhances impacts of hypoxia on oyster reefs. *Ecol Appl* 8**:**128-140.

9. Renegar, D. A. & Riegl, B. M. (2005) Effect of nutrient enrichment and elevated CO2 partial pressure on growth rate of Atlantic scleractinian coral Acropora cervicornis. *Mar Ecol Prog Ser* 293**:**69-76.

10. Bruno, J. F., Petes, L. E., Harvell, C. D. & Hettinger, A. (2003) Nutrient enrichment can increase the severity of coral diseases. *Ecol Lett* 6**:**1056-1061.

11. Voss, J. D. & Richardson, L. L. (2006) Nutrient enrichment enhances black band disease progression in corals. *Coral Reefs* 25**:**569-576.

12. Day, R. H., Doyle, T. W. & Draugelis-Dale, R. O. (2006) Interactive effects of substrate, hydroperiod, and nutrients on seedling growth of Salix nigra and Taxodium distichum. *Environ Exp Bot* 55**:**163-174.

13. Lovelock, C., Feller, I., Ellis, J., Schwarz, A., Hancock, N. & Sorrell, B. (2007) Mangrove growth in New Zealand estuaries:the role of nutrient enrichment at sites with contrasting rates of sedimentation. *Oecologia* 153**:**633-641.

14. Schloder, C. & D'Croz, L. (2004) Responses of massive and branching coral species to the combined effects of water temperature and nitrate enrichment. *J Exp Mar Biol Ecol* 313**:**255-268.

15. Nordemar, I., Nystrom, M. & Dizon, R. (2003) Effects of elevated seawater temperature and nitrate enrichment on the branching coral Porites cylindrica in the absence of particulate food. *Mar Biol* 142**:**669-677.

16. Lotze, H. K. & Worm, B. (2002) Complex interactions of climatic and ecological controls on macroalgal recruitment. *Limnol Oceanogr* 47**:**1734-1741.

17. Hagerthey, S. E., Defew, E. C. & Paterson, D. M. (2002) Influence of Corophium volutator and Hydrobia ulvae on intertidal benthic diatom assemblages under different nutrient and temperature regimes. *Mar Ecol Prog Ser* 245**:**47-59.

18. Lenihan, H. S., Peterson, C. H., Kim, S. L., Conlan, K. E., Fairey, R., McDonald, C., Grabowski, J. H. & Oliver, J. S. (2003) Variation in marine benthic community composition allows discrimination of multiple stressors. *Mar Ecol Prog Ser* 261**:**63-73.

19. Breitburg, D. L., Sanders, J. G., Gilmour, C. C., Hatfield, C. A., Osman, R. W., Riedel, G. F., Seitzinger, S. B. & Sellner, K. G. (1999) Variability in responses to nutrients and trace elements, and transmission of stressor effects through an estuarine food web. *Limnol Oceanogr* 44**:**837-863.

20. Sundback, K., Petersen, D. G., Dahllof, I. & Larson, F. (2007) Combined nutrient-toxicant effects on a shallow-water marine sediment system: sensitivity and resilience of ecosystem functions. *Mar Ecol Prog Ser* 330**:**13-30.

21. Longhi, M. L., Ferreyra, G., Schloss, I. & Roy, S. (2006) Variable phytoplankton response to enhanced UV-B and nitrate addition in mesocosm experiments at three latitudes (Canada, Brazil and Argentina). *Mar Ecol Prog Ser* 313**:**57-72.

22. Wulff, A., Wangberg, S. A., Sundback, K., Nilsson, C. & Underwood, G. J. C. (2000) Effects of UVB radiation on a marine microphytobenthic community growing on a sand-substratum under different nutrient conditions. *Limnol Oceanogr* 45**:**1144-1152.

23. Effler, R. S. & Goyer, R. A. (2006) Baldcypress and water tupelo sapling response to multiple stress agents and reforestation implications for Louisiana swamps. *For Ecol Manage* 226**:**330-340.

24. Bouma, T. J., Koutstaal, B. P., van Dongen, M. & Nielsen, K. L. (2001) Coping with low nutrient availability and inundation: root growth responses of three halophytic grass species from different elevations along a flooding gradient. *Oecologia* 126**:**472-481.

25. Torquemada, Y. F., Durako, M. J. & Lizaso, J. L. S. (2005) Effects of salinity and possible interactions with temperature and pH on growth and photosynthesis of Halophila johnsonii Eiseman. *Mar Biol* 148**:**251-260.

26. Lenssen, G. M., Vanduin, W. E., Jak, P. & Rozema, J. (1995) The Response of Aster-Tripolium and Puccinellia-Maritima to Atmospheric Carbon-Dioxide Enrichment and Their Interactions with Flooding and Salinity. *Aquat Bot* 50**:**181-192.

27. Lenssen, G. M., Lamers, J., Stroetenga, M. & Rozema, J. (1993) Interactive Effects of Atmospheric Co2 Enrichment, Salinity and Flooding on Growth of C-3 (Elymus-Athericus) and C-4 (Spartina-Anglica) Salt-Marsh Species. *Vegetatio* 104**:**379-388.

28. Kamer, K. & Fong, P. (2001) Nitrogen enrichment ameliorates the negative effects of reduced salinity on the green macroalga Enteromorpha intestinalis. *Mar Ecol Prog Ser* 218**:**87-93.

29. van Katwijk, M. M., Schmitz, G. H. W., Gasseling, A. P. & van Avesaath, P. H. (1999) Effects of salinity and nutrient load and their interaction on Zostera marina. *Mar Ecol Prog Ser* 190**:**155-165.

30. Rejmankova, E. & Komarkova, J. (2005) Response of cyanobacterial mats to nutrient and salinity changes. *Aquat Bot* 83**:**87-107.

31. Sousa, A. I., Martins, I., Lillebo, A. I., Flindt, M. R. & Pardal, M. A. (2007) Influence of salinity, nutrients and light on the germination and growth of Enteromorpha sp spores. *J Exp Mar Biol Ecol* 341**:**142-150.

32. Baldwin, A. H. & Mendelssohn, I. A. (1998) Effects of salinity and water level on coastal marshes: an experimental test of disturbance as a catalyst for vegetation change. *Aquat Bot* 61**:**255-268.

33. Howard, R. J. & Mendelssohn, I. A. (2000) Structure and composition of oligohaline marsh plant communities exposed to salinity pulses. *Aquat Bot* 68**:**143-164.

34. Webb, E. C. & Mendelssohn, I. A. (1996) Factors affecting Vegetation dieback of an oligohaline marsh in coastal Louisiana: Field manipulation of salinity and submergence. *Am J Bot* 83**:**1429-1434.

35. Howard, R. J. & Rafferty, P. S. (2006) Clonal variation in response to salinity and flooding stress in four marsh macrophytes of the northern gulf of Mexico, USA. *Environ Exp Bot* 56**:**301-313.

36. Carvalho, L. M., Correia, P. M., Cacador, I. & Martins-Loucao, M. A. (2003) Effects of salinity and flooding on the infectivity of salt marsh arbuscular mycorrhizal fungi in Aster tripolium L. *Biol Fertility Soils* 38**:**137-143.

37. Charpentier, A., Mesleard, F. & Grillas, P. (1998) The role of water level and salinity in the regulation of Juncus gerardi populations in former ricefields in southern France. *J Veg Sci* 9**:**361-370.

38. Hellings, S. E. & Gallagher, J. L. (1992) The Effects of Salinity and Flooding on Phragmites-Australis. *J Appl Ecol* 29**:**41-49.

39. Naidoo, G. & Kift, J. (2006) Responses of the saltmarsh rush Juncus kraussii to salinity and waterlogging. *Aquat Bot* 84**:**217-225.

40. Koch, M. S. & Erskine, J. M. (2001) Sulfide as a phytotoxin to the tropical seagrass Thalassia testudinum: interactions with light, salinity and temperature. *J Exp Mar Biol Ecol* 266**:**81-95.

41. Gama-Flores, J. L., Sarma, S. S. S. & Nandini, S. (2005) Interaction among copper toxicity, temperature and salinity on the population dynamics of Brachionus rotundiformis (Rotifera). *Hydrobiologia* 546**:**559-568.

42. Przeslawski, R., Davis, A. R. & Benkendorff, K. (2005) Synergistic effects associated with climate change and the development of rocky shore molluscs. *Global Change Biol* 11**:**515-522.

43. Ushakova, O. O. (2003) Combined effect of salinity and temperature on Spirorbis spirorbis L. and Circeus spirillum L. larvae from the White Sea. *J Exp Mar Biol Ecol* 296**:**23-33.

44. Qiu, J. W. & Qian, P. Y. (1998) Combined effects of salinity and temperature on juvenile survival, growth and maturation in the polychaete Hydroides elegans. *Mar Ecol Prog Ser* 168**:**127-134.

45. Nielsen, M. V. & Tonseth, C. P. (1991) Temperature and Salinity Effect on Growth and Chemical-Composition of Gyrodinium-Aureolum Hulburt in Culture. *J Plankton Res* 13**:**389-398.

46. Kwok, K. W. H. & Leung, K. M. Y. (2005) Toxicity of antifouling biocides to the intertidal harpacticoid copepod Tigriopus japonicus (Crustacea, Copepoda): Effects of temperature and salinity. *Mar Pollut Bull* 51**:**830-837.

47. Brenchley, J. L. & Probert, R. J. (1998) Seed germination responses to some environmental factors in the seagrass Zostera capricorni from eastern Australia. *Aquat Bot* 62**:**177-188.

48. Alutoin, S., Boberg, J., Nystrom, M. & Tedengren, M. (2001) Effects of the multiple stressors copper and reduced salinity on the metabolism of the hermatypic coral Porites lutea. *Mar Environ Res* 52**:**289-299.

49. Elfwing, T. & Tedengren, M. (2002) Effects of copper and reduced salinity on grazing activity and macroalgae production: a short-term study on a mollusc grazer, Trochus maculatus and two species of macroalgae in the inner Gulf of Thailand. *Mar Biol* 140**:**913-919.

50. De Lisle, P. F. & Roberts, M. H. (1994) The Effect of Salinity on Cadmium Toxicity in the Estuarine Mysid Mysidopsis-Bahia - Roles of Osmoregulation and Calcium. *Mar Environ Res* 37**:**47-62.

51. Karsten, U., Dummermuth, A., Hoyer, K. & Wiencke, C. (2003) Interactive effects of ultraviolet radiation and salinity on the ecophysiology of two Arctic red algae from shallow waters. *Polar Biol* 26**:**249-258.

52. Kahn, A. E. & Durako, M. J. (2006) Thalassia testudinum seedling responses to changes in salinity and nitrogen levels. *J Exp Mar Biol Ecol* 335**:**1-12.

53. Porter, J. W., Lewis, S. K. & Porter, K. G. (1999) The effect of multiple stressors on the Florida Keys coral reef ecosystem: A landscape hypothesis and a physiological test. *Limnol Oceanogr* 44**:**941-949.

54. Piazzi, L., Balata, D., Ceccherelli, G. & Cinelli, F. (2005) Interactive effect of sedimentation and Caulerpa racemosa var. cylindracea invasion on macroalgal assemblages in the Mediterranean Sea. *Estuarine Coastal and Shelf Science* 64**:**467-474.

55. Gorgula, S. K. & Connell, S. D. (2004) Expansive covers of turf-forming algae on human-dominated coast: the relative effects of increasing nutrient and sediment loads. *Mar Biol* 145**:**613-619.

56. Gray, A. J. & Mogg, R. J. (2001) Climate impacts on pioneer saltmarsh plants. *Clim Res* 18**:**105-112.

57. Reynaud, S., Leclercq, N., Romaine-Lioud, S., Ferrier-Pages, C., Jaubert, J. & Gattuso, J. P. (2003) Interacting effects of CO2 partial pressure and temperature on photosynthesis and calcification in a scleractinian coral. *Global Change Biol* 9**:**1660-1668.

58. Fu, F. X., Warner, M. E., Zhang, Y. H., Feng, Y. Y. & Hutchins, D. A. (2007) Effects of increased temperature and CO2 on photosynthesis, growth, and elemental ratios in marine Synechococcus and Prochlorococcus (Cyanobacteria). *J Phycol* 43**:**485-496.

59. Cervino, J. M., Hayes, R. L., Polson, S. W., Polson, S. C., Goreau, T. J., Martinez, R. J. & Smith, G. W. (2004) Relationship of Vibrio species infection and elevated temperatures to yellow blotch/band disease in Caribbean corals. *Appl Environ Microbiol* 70**:**6855-6864.

60. Ward, J. R., Kim, K. & Harvell, C. D. (2006) Temperature affects coral disease resistance and pathogen growth. *Mar Ecol Prog Ser* 329**:**115-121.

61. Braid, B. A., Moore, J. D., Robbins, T. T., Hedrick, R. P., Tjeerdema, R. S. & Friedman, C. S. (2005) Health and survival of red abalone, Haliotis rufescens, under varying temperature, food supply, and exposure to the agent of withering syndrome. *J Invertebr Pathol* 89**:**219-231.

62. Aeby, G. S. & Santavy, D. L. (2006) Factors affecting susceptibility of the coral Montastraea faveolata to black-band disease. *Mar Ecol Prog Ser* 318**:**103-110.

63. Mora, C., Metzger, R., Rollo, A. & Myers, R. (2007) Experimental simulations about the effects of overexploitation and habitat fragmentation on populations facing environmental warming. *Proc R Soc Lond, Ser B: Biol Sci* 274 1023 - 1028.

64. Cancino, J. M., Gallardo, J. A. & Torres, F. A. (2003) Combined effects of dissolved oxygen concentration and water temperature on embryonic development and larval shell secretion in the marine snail Chorus giganteus (Gastropoda : Muricidae). *Mar Biol* 142**:**133-139.

65. Hicks, D. W. & McMahon, R. F. (2005) Effects of temperature on chronic hypoxia tolerance in the non-indigenous brown mussel, Perna perna (Bivalvia : Mytilidae) from the Texas Gulf of Mexico. *J Molluscan Stud* 71**:**401-408.

66. Vilchis, L. I., Tegner, M. J., Moore, J. D., Friedman, C. S., Riser, K. L., Robbins, T. T. & Dayton, P. K. (2005) Ocean warming effects on growth, reproduction, and survivorship of Southern California abalone. *Ecol Appl* 15**:**469-480.

67. Anthony, K. R. N., Connolly, S. R. & Hoegh-Guldberg, O. (2007) Bleaching, energetics, and coral mortality risk: Effects of temperature, light, and sediment regime. *Limnol Oceanogr* 52**:**716-726.

68. Nystrom, M., Nordemar, I. & Tedengren, M. (2001) Simultaneous and sequential stress from increased temperature and copper on the metabolism of the hermatypic coral Porites cylindrica. *Mar Biol* 138**:**1225-1231.

69. Drohan, A. F., Thoney, D. A. & Baker, A. C. (2005) Synergistic effect of high temperature and ultraviolet-B radiation on the gorgonian Eunicea tourneforti (Octocorallia : Alcyonacea : Plexauridae). *Bull Mar Sci* 77**:**257-266.

70. Rautenberger, R. & Bischof, K. (2006) Impact of temperature on UV-susceptibility of two Ulva (Chlorophyta) species from Antarctic and Subantarctic regions. *Polar Biol* 29**:**988-996.

71. Lesser, M. P. (1996) Elevated temperatures and ultraviolet radiation cause oxidative stress and inhibit photosynthesis in symbiotic dinoflagellates. *Limnol Oceanogr* 41**:**271-283.

72. Hoffman, J. R., Hansen, L. J. & Klinger, T. (2003) Interactions between UV radiation and temperature limit inferences from single-factor experiments. *J Phycol* 39**:**268-272.

73. Altamirano, M., Flores-Moya, A. & Figueroa, F. L. (2003) Effects of UV radiation and temperature on growth of germlings of three species of Fucus (Phaeophyceae). *Aquat Bot* 75**:**9-20.

74. Ferrier-Pages, C., Richard, C., Forcioli, D., Allemand, D., Pichon, M. & Shick, J. M. (2007) Effects of temperature and UV radiation increases on the photosynthetic efficiency in four scleractinian coral species. *Biological Bulletin* 213**:**76-87.

75. Lesser, M. P., Stochaj, W. R., Tapley, D. W. & Shick, J. M. (1990) Bleaching in Coral-Reef Anthozoans - Effects of Irradiance, Ultraviolet-Radiation, and Temperature on the Activities of Protective Enzymens against Active Oxygen. *Coral Reefs* 8**:**225-232.

76. Anderson, R. S., Brubacher, L. L., Calvo, L. R., Unger, M. A. & Burreson, E. M. (1998) Effects of tributyltin and hypoxia on the progression of Perkinsus marinus infections and host defence mechanisms in oyster, Crassostrea virginica (Gmelin). *J Fish Dis* 21**:**371-379.

77. Koch, M. S., Schopmeyer, S., Kyhn-Hansen, C. & Madden, C. J. (2007) Synergistic effects of high temperature and sulfide on tropical seagrass. *J Exp Mar Biol Ecol* 341**:**91-101.

78. Liess, M., Champeau, O., Riddle, M., Schulz, R. & Duquesne, S. (2001) Combined effects of ultraviolet-B radiation and food shortage on the sensitivity of the Antarctic amphipod Paramoera walkeri to copper. *Environ Toxicol Chem* 20**:**2088-2092.

79. Sargian, P., Mostajir, B., Chatila, K., Ferreyra, G. A., Pelletier, E. & Demers, S. (2005) Non-synergistic effects of water-soluble crude oil and enhanced ultraviolet-B radiation on a natural plankton assemblage. *Mar Ecol Prog Ser* 294**:**63-77.

80. Sargian, P., Pelletier, E., Mostajir, B., Ferreyra, G. A. & Demers, S. (2005) TBT toxicity on a natural planktonic assemblage exposed to enhanced ultraviolet-B radiation. *Aquat Toxicol* 73**:**299-314.

81. Duquesne, S. & Liess, M. (2003) Increased sensitivity of the macroinvertebrate Paramorea walkeri to heavy-metal contamination in the presence of solar UV radiation in Antarctic shoreline waters. *Mar Ecol Prog Ser* 255**:**183-191.

82. Peachey, R. B. J. (2005) The synergism between hydrocarbon pollutants and UV radiation: a potential link between coastal pollution and larval mortality. *J Exp Mar Biol Ecol* 315**:**103-114.

83. Martinez, M. D. G., Romero, P. R. & Banaszak, A. T. (2007) Photoinduced toxicity of the polycyclic aromatic hydrocarbon, fluoranthene, on the coral, Porites divaricata. *Journal of Environmental Science and Health Part a-Toxic/Hazardous Substances & Environmental Engineering* 42**:**1495-1502.

84. Cleveland, L., Little, E. E., Calfee, R. D. & Barron, M. G. (2000) Photoenhanced toxicity of weathered oil to Mysidopsis bahia. *Aquat Toxicol* 49**:**63-76.

85. Pelletier, E., Sargian, P., Payet, J. & Demers, S. (2006) Ecotoxicological effects of combined UVB and organic contaminants in coastal waters: A review. *Photochem Photobiol* 82**:**981-993.

86. Little, E. E., Cleveland, L., Calfee, R. & Barron, M. G. (2000) Assessment of the photoenhanced toxicity of a weathered oil to the tidewater silverside. *Environ Toxicol Chem* 19**:**926-932.

87. Sargian, P., Mas, S., Pelletier, E. & Demers, S. (2007) Multiple stressors on an Antarctic microplankton assemblage: water soluble crude oil and enhanced UVBR level at Ushuaia (Argentina). *Polar Biol* 30**:**829-841.

88. Southerland, H. A. & Lewitus, A. J. (2004) Physiological responses of estuarine phytoplankton to ultraviolet light-induced fluoranthene toxicity. *J Exp Mar Biol Ecol* 298**:**303-322.

89. Steevens, J. A., Slattery, M., Schlenk, D., Aryl, A. & Benson, W. H. (1999) Effects of ultraviolet-B light and polyaromatic hydrocarbon exposure on sea urchin development and bacterial bioluminescence. *Mar Environ Res* 48**:**439-457.

90. Miller, M. W. & Hay, M. E. (1996) Coral-seaweed-grazer-nutrient interactions on temperate reefs *Ecol. Monogr.* **66,** 323-344.

91. McClanahan, T. R., Sala, E., Stickels, P. A., Cokos, B. A., Baker, A. C., Starger, C. J. & Jones, S. H. (2003) Interaction between nutrients and herbivory in controlling algal communities and coral condition on Glover's Reef, Belize *Marine Ecology-Progress Series* **261,** 135-147.

92. Thacker, R. W., Ginsburg, D. W. & Paul, V. J. (2001) Effects of herbivore exclusion and nutrient enrichment on coral reef macroalgae and cyanobacteria *Coral Reefs* **19,** 318-329.
